# Identification and proteomic profiling of exosomes in human cerebrospinal fluid

**DOI:** 10.1186/1479-5876-10-5

**Published:** 2012-01-05

**Authors:** Jonathan M Street, Perdita E Barran, C Logan Mackay, Stefan Weidt, Craig Balmforth, Tim S Walsh, Rod TA Chalmers, David J Webb, James W Dear

**Affiliations:** 1 University/British Heart Foundation Centre for Cardiovascular Science, Queen's Medical Research Institute, The University of Edinburgh, Edinburgh, UK; 2 EastChem School of Chemistry, The University of Edinburgh, Edinburgh, UK; 3 Royal Infirmary of Edinburgh, Critical Care, Edinburgh, UK; 4 Royal Infirmary of Edinburgh, Vascular Surgery Service, Edinburgh, UK

**Keywords:** Exosomes, human, CSF, proteomics

## Abstract

**Background:**

Exosomes are released from multiple cell types, contain protein and RNA species, and have been exploited as a novel reservoir for disease biomarker discovery. They can transfer information between cells and may cause pathology, for example, a role for exosomes has been proposed in the pathophysiology of Alzheimer's disease. Although studied in several biofluids, exosomes have not been extensively studied in the cerebrospinal fluid (CSF) from humans. The objective of this study was to determine: 1) whether human CSF contains exosomes and 2) the variability in exosomal protein content across individuals.

**Methods:**

CSF was collected from 5 study participants undergoing thoraco-abdominal aortic aneurysm repair (around 200 - 500 ml per participant) and low-density membrane vesicles were concentrated by ultracentrifugation. The presence of exosomes was determined by western blot for marker proteins, isopycnic centrifugation on a sucrose step gradient and transmission electron microscopy with immuno-labelling. Whole protein profiling was performed using Fourier transform ion cyclotron resonance mass spectrometry (FT-ICR).

**Results:**

Flotillin 1 and tumor susceptibility gene 101 (TSG101), two exosomal marker proteins, were identified in the ultracentrifugation pellet using western blot. These markers localized to a density consistent with exosomes following isopycnic centrifugation. Transmission electron microscopy visualized structures consistent with exosomes in size and appearance that labelled positive for flotillin 1. Therefore, the pellet that resulted from ultracentrifugation of human CSF contained exosomes. FT-ICR profiling of this pellet was performed and 84-161 ions were detected per study participant. Around one third of these ions were only present in a single study participant and one third were detected in all five. With regard to ion quantity, the median coefficient of variation was 81% for ions detected in two or more samples.

**Conclusions:**

Exosomes were identified in human CSF and their proteome is a potential new reservoir for biomarker discovery in neurological disorders such as Alzheimer's disease. However, techniques used to concentrate exosomes from CSF need refinement to reduce variability. In this study we used relatively large starting volumes of human CSF, future studies will focus on exosome isolation from smaller 'real life' clinical samples; a key challenge in the development of exosomes as translational tools.

## Background

Exosomes are lipid and protein rich vesicles that are formed as part of the intra-cellular endosomal pathway [1]. During maturation of early endosomes into late endosomes within the cell, the endosomal limiting membrane undergoes invagination forming intra-luminal vesicles. A subset of endosomes fuse with the plasma membrane and their intra-luminal vesicles are released into the extracellular space where they are termed exosomes. Exosomes have physicochemical properties that distinguish them from other cell-derived vesicles. They are 20-100 nm in size [2], appear cup-shaped when visualised by transmission electron microscopy (TEM) [3], have a density of 1.10 to 1.19 g/ml [4,5] and contain characteristic proteins that are central to their production [6]. Such proteins include flotillin-1, which is associated with lipid rafts that act as the location for exosomal formation [6] and tumor susceptibility gene 101 (TSG101), a component of the Endosomal Sorting Complex Required for Transport (ESCRT) protein group that mediates exosome assembly [7].

Exosomes represent a novel reservoir for biomarker discovery because they contain protein, messenger RNA and microRNA that has been demonstrated to change with the disease state of the affected organ [8,9]. To date, in the nervous system, most research into exosomes has been *in vitro*, for example, primary cultures of rat cortical neurons [10], differentiated cortical neurons [11] and glial cells [12,13] have been demonstrated to release exosomes. Using TEM and laser correlation spectroscopy, structures with a similar morphology to exosomes have been reported in human cerebrospinal fluid (CSF) [14,15]. The centrifugation fraction that expressed these structures on TEM contained the ras-related protein Ral-A, a protein that has been associated with exosomes [14,16].

In addition to being a source of biomarkers there is evidence that exosomes may mediate neurological disease. In Alzheimer's disease (AD), cleavage of amyloid precursor protein by the enzyme β-secretase occurs in a subset of endosomes and a fraction of the Aβ protein is released into the extracellular milieu in association with exosomes [17,18]. Furthermore, exosomal markers are enriched in amyloid plaques from the brains of mice [19] and the post-mortem brains of patients with AD [17]. This suggests that exosomal transfer of amyloid into the extra-cellular space may be an important pathway in the development of AD. Extracellular accumulation of tau protein is another pathological hallmark of AD. This protein is secreted in exosomes and an elevated CSF tau concentration in patients with AD may be due to exosomal release from cells [20]. The protein α-synuclein is a mediator of neurodegeneration in Parkinson's disease (PD) that is released from cultured cells in exosomes [21]. Application of α-synuclein containing exosomes onto cells induced cell death; consistent with exosomes being mediators of inter-cellular signalling, a physiological mechanism that has been reported in other organs [22,23]. Prion diseases are infectious neurodegenerative disorders and there is *in vitro *evidence that exosomes may represent the mechanism of prion protein transfer between cells [24,25]. In sheep CSF, prion proteins are enriched in exosomes [26]. Given their potential as a reservoir for biomarker discovery, and as mediators of neurological disease, the first objective of the present study was to confirm that human CSF contains exosomes.

The protein composition of a complex biofluid can be investigated using mass spectrometry [27-30]. This approach allows for an unbiased assessment of the proteome without the need for prior knowledge of protein identities. At present, there is no method for isolating exosomes from a complex biofluid without a degree of 'contamination' from non-exosomal proteins. Using quantitative mass spectrometry the second objective was to profile the composition of the human CSF sub-fraction that contained exosomes to give an indication of the variability and so provide information for planning future biomarker discovery studies.

## Methods

### Study participants and sample collection

CSF was collected from 5 patients undergoing thoraco-abdominal aortic aneurysm repair. None of the patients had a history of neurological illness. This study was approved by NHS Lothian research ethics committee and prospective informed consent was obtained from all patients. This patient group was chosen because they have a CSF drain inserted peri-operatively as part of routine clinical management and large volumes of CSF are available for study (around 200 - 500 ml). Men were recruited to this study because female sex hormones may alter exosome release. The CSF was carefully inspected for evidence of blood contamination before collection. Protease inhibitors and preservative (final concentration: 3.34 mM sodium azide, 0.5 mM phenylmethylsulphonyl fluoride, 20 μM leupeptin) were added to the collected CSF. The collected CSF was stored in aliquots at -80°C before use [31].

### Exosome concentration

After thawing, the CSF was vigorously vortexed then centrifuged at 15,000 × *g *for 10 minutes to pellet any shed cells, large membrane fragments and other debris. The supernatant was then centrifuged at 200,000 × *g *for 60 minutes. The pellet was resuspended in phosphate buffered saline (PBS) and then re-centrifuged at 200,000 × *g *for 60 minutes before final resuspension in PBS. The protein content of the exosomal fraction was determined using the BCA protein assay kit (Pierce, Rockford, IL, USA).

### Western blotting

Samples were solubilised with Laemmli sample buffer and separated on a 1D SDS-PAGE gel before transfer to polyvinylidene fluoride (PVDF, Invitrogen, Paisley, UK) membrane. The membrane was probed with the following primary antibodies: mouse anti-human flotillin-1 (BD Biosciences, Franklin Lakes, USA) and mouse anti-human TSG101 (Abcam, Cambridge, UK).

### Sucrose density gradient

Sample was diluted into the top fraction of a step gradient comprising layers of 2, 1.3, 1.16, 0.8, 0.5 and 0.25 M sucrose. The gradients were centrifuged for 2.5 hours at 100,000 × *g*. Six fractions were collected from the gradient and stored for density determination on a DMA 35 N density meter (Anton Paar, Hertford, UK) and for western blot analysis.

### Electron microscopy

The exosomal fraction was mixed 1:1 with 4% paraformaldehyde. A drop of this solution was then placed on a Petri dish and a formvar-coated 200-mesh gold grid floated on top for 20 minutes. The grid was washed in PBS before low and high molecular weight blocking using 0.05 M glycine/PBS and 1% BSA/PBS, respectively. The grid was re-washed and transferred to a 1:200 dilution of the anti-flotillin-1 antibody or the isotype control in 0.02% Triton X-100 PBS for 45 minutes at room temperature. The grid was then incubated with a 10 nm gold conjugated anti-mouse IgG antibody (Sigma Aldrich, Gillingham, UK) for 60 minutes at room temperature. The grid was washed again and incubated with 1% glutaraldehyde for 5 minutes. The grid was washed and then contrasted and embedded with 0.5% uranyl acetate/2% methyl cellulose. Excess fluid was removed, the grid allowed to air dry and then the grid was examined on a Philips CM120 BioTwin transmission electron microscope.

### Proteomic analysis

Proteomic studies were performed on the ultra-centrifugation pellet from each study participant, with each pellet being analysed in duplicate as per previous studies [32]. Initial studies demonstrated a high concentration of immunoglobulins in CSF. Therefore, after ultra-centrifugation, the immunoglobulins were depleted from the samples by incubation with protein G agarose beads (Fluka, Gillingham, UK). Depletion of immunoglobulin was confirmed by western blotting using an anti immunoglobulin primary antibody (AbD Serotec, Kidlington, UK). After immunoglobulin depletion, the presence of exosomes was confirmed by western blotting for flotillin-1.

### FT-ICR mass spectrometry

The proteins present in the ultra-centrifugation pellet were separated from the lipid components using a chloroform/methanol precipitation [33]. Following resuspension of the chloroform/methanol precipitation pellet, separation was on a UltiMate 3000 series HPLC instrument (Dionex, Sunnyvale, CA, USA) equipped with a PS-DVB monolithic column (500 μm × 5 cm). Mass spectra were acquired on a 12T Apex Ultra Qe FT-ICR mass spectrometer (Bruker Daltonics, Billerica, MA, USA), equipped with a electrospray source. Instrument control was achieved using Apex control. Each analysis was the average of 2 spectra. Ions were detected between *m/z *200 and 3000, yielding 1 Mword time-domain transients.

The mass spectrometry data was processed using the DataAnalysis (Bruker Daltonics, Billerica, MA, USA) software package version 4 SP4. The Progenesis LC-MS v2.6 software package (Non-linear Dynamics, Newcastle upon Tyne, UK) was used for the quantitative mass spectrometry. This package matched the peaks from the ion chromatograms of our 5 study participants by the elution time and m/z ratio. The quality of the matching was checked manually. This matching process allowed determination of the number of samples which contained a given protein or peptide. The ion current was integrated over the elution window, then normalised against the total ion current of the chromatogram, and comparative quantitation of the abundance of an ion detected in 2 or more samples was calculated. Data analysis was performed in the python language with the Scipy http://www.scipy.org, and Matplotlib [34] packages used for general statistical analysis and data visualisation respectively.

## Results

**Figure 1:**
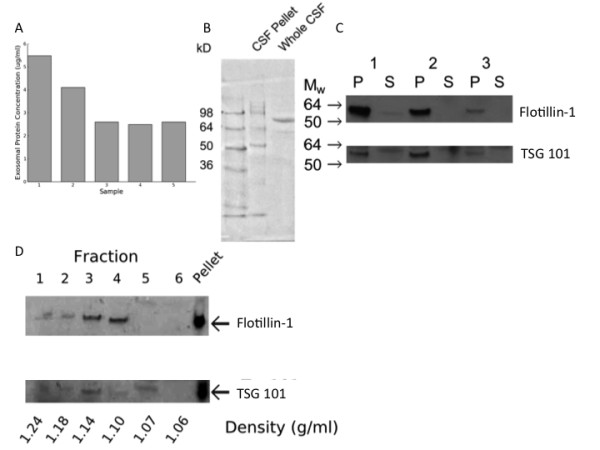
**A) The pellet resulting from ultracentrifugation of human CSF contained protein**. The amount of protein is expressed as per ml of CSF. Samples 1 - 5 are from 5 different study participants. B) The size distribution of protein was different in the pellet resulting from ultracentrifugation of human CSF (CSF pellet) compared with uncentrifuged CSF (whole CSF). C) Western blots for flotillin-1 and TSG-101 on the ultracentrifugation pellet (P) and supernatant (S) from CSF collected from 3 study participants (1-3). Both exosomal markers were enriched in the pellets in comparison to the supernatants. D) Western blot for flotillin-1 and TSG101 on fractions obtained following isopycnic centrifugation. The exosomal markers were present in fractions corresponding to a density of 1.10-1.14 g.cm^-3 ^.

CSF was collected from 5 patients whose demographics are presented in Table 1. Ultracentrifugation of human CSF formed a pellet that contained protein (Figure 1a). Following separation of the proteins in the pellet there were bands unique to the ultracentrifuge pellet sample, suggesting that this may be a distinct proteome (Figure 1b). To investigate whether this proteome was, at least in part, exosomal in origin, the presence of established exosomal markers was determined using western blot for flotillin-1 and TSG101. This demonstrated enrichment of both proteins in the pellet compared with unfractionated CSF (Figure 1c). To further confirm that flotillin-1 and TSG101 were located in exosomes, isopycnic centrifugation on a sucrose step gradient was performed. Flotillin-1 and TSG101 localised to fractions 3 and 4, which corresponded to a density of 1.10-1.14 g.cm^3 ^(Figure 1d). Isopycnic centrifugation was performed in 4 subjects and the exosomal markers were consistently localized to the density range 1.10-1.18 g.cm**^-3 ^**(data not shown).

**Table 1:** Demographics and comorbidities for the 5 patients.

Patient	Sex	Age	Comorbidity
1	M	67	IHD and hypotension

2	M	72	Hypertension and CKD

3	M	77	Hypertension

4	M	70	DM and IHD

5	M	68	None

Abbreviations; IHD, Ischaemic heart disease; CKD, Chronic kidney disease; DM, Diabetes mellitus.

**Figure 2:**
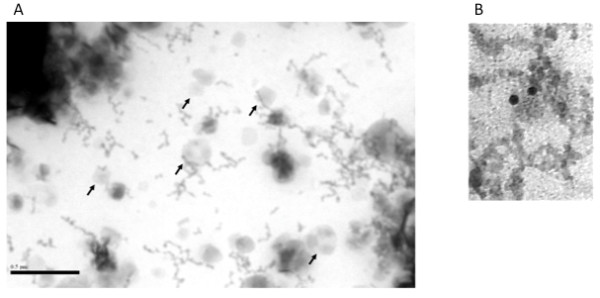
**A) Transmission electron micrographs of embedded and negatively stained human CSF**. Several structures of the characteristic size and appearance for exosomes are highlighted by arrows. B) Transmission electron micrographs of human CSF exosomes incubated with an anti-flotillin-1 antibody and then a gold nanoparticle tagged secondary antibody. A structure with the appearance of an exosome labelled by 2 gold nanoparticles is demonstrated in the figure.

TEM revealed a number of structures of exosomal size and appearance (Figure 2a) but there were also structures of a larger size, as has been previously reported [14]. To confirm that exosomes were present, labelling with an anti-flotillin 1 antibody was performed and cup-shaped structures of the characteristic size for exosomes were found to bind gold nanoparticles (Figure 2b). When the flotillin-1 antibody was replaced with an isotype control antibody, although structures of the characteristic size and appearance could be identified, a significantly reduced number of bound gold nanoparticles were present (gold nanoparticle binding (mean ± SD): flotillin-1 antibody 0.6 ± 0.4 particles per exosome; isotype control 0.1 ± 0.1 particles per exosome, p = 0.006 by t-test).

**Figure 3:**
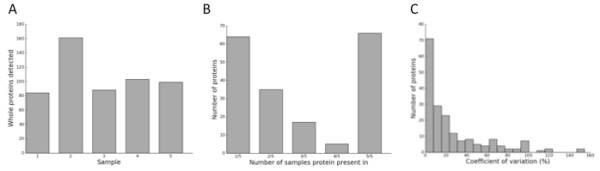
**Ions detected following FT-ICR MS on the pellet that resulted from ultracentrifugation of human CSF**. A) The number of ions detected in each sample. Sample 1 - 5 are from 5 different study participants B) The number of ions detected in multiple samples. C) Histogram for the coefficient of variation of ions detected in 2 or more of the 5 CSF ultracentrifugation pellets.

Profiling using FT-ICR MS detected between 84-161 ions in the 5 ultracentrifugation pellets (Figure 3a). The range of molecular weights were 636-33467 Da. Coefficient of variation (CV) for the retention time was 0.13%, facilitating the automated alignment performed by the Progenesis software. 66 ions were present in all 5 participants and 64 ions were only found in a single study participant (Figure 3b). For ions detected in two or more samples, the variability in the integrated ion current (a measure of an ion's abundance in a particular sample) across study participants was 68% at the 25^th ^percentile (25% of ions had a CV < 68%) and the median was 81% (Figure 3c). Among the ten most abundant ions the maximum variation was 13.9% (Table 2). Additional File 1 contains the whole dataset from FT-ICR MS.

**Table 2:** The ten most abundant ions in the pellet that resulted from ultracentrifugation of human CSF as measured by FT-ICR MS.

m/z	Charge	Average Abundance	Coefficient of variation (%)	Retention Time (min)				
				
				Retention 1	Retention 2	Retention 3	Retention 4	Retention 5
659.5042	2	84905.83	9.48	32.35	32.34	32.44	32.41	32.39

637.4901	2	79016.43	8.88	32.35	32.34	32.44	32.41	32.39

681.5181	2	73538.83	10.40	32.35	32.34	32.44	32.41	32.39

615.4762	2	71518.13	7.83	32.28	32.28	32.38	32.35	32.33

703.5339	2	70627.72	10.75	32.42	32.40	32.51	32.47	32.44

593.462	2	64351.47	9.18	32.28	32.28	32.38	32.35	32.33

571.4479	2	51233.77	10.47	32.22	32.21	32.31	32.29	32.28

725.5471	2	45044.52	9.20	32.42	32.40	32.51	32.47	32.44

549.4341	2	41728.51	8.51	32.22	32.21	32.31	32.29	32.28

527.42	2	29260.44	13.92	32.15	32.15	32.24	32.23	32.22

## Discussion

Release of exosomes from a variety of different neuronal cell lines has been described [10] and there is evidence that exosomes may be involved in the pathogenesis of neurological disease [17,18]. Despite the potential importance of exosomes in the context of the central nervous system and neurological diseases the presence of exosomes in human CSF has not been confirmed. In the present study we have demonstrated that exosomes are present in the CSF of humans. The evidence for exosome presence takes three forms: 1) proteins widely documented to be present within exosomes are enriched in the ultracentrifugation pellet in comparison with the supernatant; 2) following separation of the ultracentrifugation pellet based on density, the markers localise to a density range which is consistent with these proteins being contained in exosomes; 3) direct visualisation of the resuspended membrane vesicles from the ultracentrifugation pellet revealed the presence of structures matching exosomes in size and shape. In a further experiment these structures were demonstrated to contain the exosomal marker flotillin-1.

Exosomes are membrane bound vesicles formed within the cellular endosomal system [1] and released into a wide range of human biofluids such as blood [35], urine [2], ascites [36], amniotic fluid [37], pleural fluid [38] and saliva [39,40]. They contain protein, messenger RNA and microRNA [41] from their cell of origin which changes with cell activation and injury [42] and represents a reservoir for disease biomarker discovery. Exosomes also mediate inter-cellular communication by transferring protein and RNA species between cells and altering the recipient cell phenotype [22,23]. For example, in the immune system, exosomes transfer microRNA from T-lymphocytes to antigen presenting cells and this microRNA modulates gene expression in the recipient cells [43]. The ability of the exosome to transfer information has been exploited to deliver systemically administered, short interfering RNA to the brain with a high degree of tissue-specificity [44]. In the present study we confirm the presence of exosomes in human CSF. This 'proof of concept' study should stimulate investigation of the differences between disease and control in terms of the exosome composition. Exosomes have been demonstrated to play a role in the pathogenesis of AD and an investigation of CSF exosomes from patients with AD is a potential next study. However, there are important caveats that need consideration before such a study can be successfully performed. In our study we recruited patients undergoing thoraco-abdominal aortic aneurysm repair because this allowed access to large CSF volumes. Such volumes will not be available from patients with AD and so there is a need to refine our techniques for exosome isolation using approaches such as isopycnic centrifugation, ultrafiltration [45] or immunoaffinity [46]. In comparison to protein, the study of RNA has the advantage that amplification techniques are available and this may allow human CSF exosomal RNA to be studied with routinely available CSF volumes. As proof of concept, exosomes in human amniotic fluid, saliva and urine have been demonstrated to contain sufficient RNA to determine the presence of single nucleotide polymorphisms [37]. Finally, as the cellular origin of the exosomes is unknown, we do not know whether the cells affected by AD actually release exosomes into human CSF.

The human CSF exosomes were concentrated by ultra-centrifugation. This technique does not completely isolate exosomes from other components of a complex biofluid and non-exosomal proteins may still be present. To reduce this contamination, immunoglobulins were depleted before analysis. Mass spectrometry based profiling of our 5 study participants was performed to begin the process of understanding the variability between ultracentrifugation pellets prepared from different people. When mass spectrometer generated ion currents are being compared, specific tags can be used to label proteins from different proteomes. The two most commonly used methods are isotope-coded affinity tags (ICAT) and isobaric tag for relative and absolute quantification (iTRAQ)[47]. Both these techniques involve additional cost, experimental steps and, potentially, the inclusion of additional errors. Also, comparison across 5 proteomes would be difficult due to limited numbers of distinct labels. Our approach was label-free, and this was possible because FT-ICR MS provided extremely high accuracy in the m/z ratios, ensuring confidence in matching ions across samples [48]. We only detected low molecular weight ions, that probably represent polypeptide species and small proteins, because larger species were deliberately excluded from entering the chromatography column. This was necessary to prevent large proteins attaching and failing to elute. Around a third of ions were present in all 5 study participants but a similar number were only detected in a single sample. For ions that were detected in 2 or more samples, the coefficients of variation were approximately comparable to similar studies including one in the whole CSF [49]. The ions that were only detected in single samples may represent technical variability in sample preparation, contamination from non-exosomal proteins or true variability in exosomal composition. Improved techniques for exosome isolation may reduce the variability in the samples. In the present study the ultra-centrifugation pellet from each study participant was analysed in duplicate, the same number of technical replicates as performed in other studies that have used mass spectrometry to quantify differences in human CSF protein abundance [32]. Increasing the number of biological and technical replicates may increase the ion coverage and improve confidence that an observed difference in an ion represents a true difference in the exosomal proteome.

## Conclusions

We have identified exosomes in the CSF from humans. These cell-derived particles represent a new reservoir for neurological disease protein and RNA biomarker discovery. However, the techniques used to concentrate exosomes from CSF need refinement to improve the quality of exosome isolation. In this study we used relatively large starting volumes of human CSF, future studies will focus on exosome isolation from smaller 'real life' clinical samples; a key challenge in the development of exosomes as translational tools.

## List of abbreviations

AD: Alzheimer's disease; CNS: Central Nervous System; CSF: Cerebrospinal fluid; ESCRT-I: Endosomal Sorting Complex Required for Transport; FT-ICR: Fourier transform ion cyclotron resonance mass spectrometry; TEM: Transmission electron microscopy; TSG101: Tumor susceptibility gene 101.

## Competing interests

The authors declare that they have no competing interests.

## Authors' contributions

JMS isolated exosomes and performed density studies, TEM and mass spectrometry with supervision of JWD (for all studies) DJW (for exosome isolation), PB (mass spectrometry), CLM (mass spectrometry) and SW (mass spectrometry). CB performed immunoblotting on exosomes. TSW and RTAC identified and recruited patients and organised sample collection. All authors read and approved the final manuscript.

## Supplementary Material

Additional file 1 **Mass Spectrometry data**. Whole dataset from FT-ICR MS on human CSF exosomes. Click here for file
