# Supplementary material for: Identification and proteomic profiling of exosomes in human cerebrospinal fluid
Source: J Transl Med. 2012 Jan 5;10:5. doi: 10.1186/1479-5876-10-5 (PMC3275480; doi:10.1186/1479-5876-10-5)
Supplement: Additional file 1 — Mass Spectrometry data. Whole dataset from FT-ICR MS on human CSF exosomes. [file 1479-5876-10-5-S1.DOC]

Additional file 1

Quantitative FT-ICR MS on CSF exosomal proteome

| **m/z** | **Retention time (min)** | **Retention time window (min)** | **Charge** | **Normalized abundance** | | | | | **Sample retention time (min)** | | | | |
| --- | --- | --- | --- | --- | --- | --- | --- | --- | --- | --- | --- | --- | --- |
|  |  |  |  | **1** | **2** | **3** | **4** | **5** | **1** | **2** | **3** | **4** | **5** |
| 659.5042 | 32.351 | 0.946 | 2 | 93615.42 | 72355.22 | 83941.47 | 89867.90 | 84749.16 | 32.351 | 32.340 | 32.443 | 32.406 | 32.387 |
| 615.4762 | 32.283 | 1.213 | 2 | 71163.90 | 62386.37 | 74296.14 | 77277.90 | 72466.33 | 32.283 | 32.276 | 32.376 | 32.346 | 32.332 |
| 637.4901 | 32.351 | 1.146 | 2 | 84422.49 | 66849.94 | 79960.09 | 82860.75 | 80988.87 | 32.351 | 32.340 | 32.443 | 32.406 | 32.387 |
| 593.462 | 32.283 | 1.079 | 2 | 63779.87 | 54740.21 | 65497.96 | 70420.12 | 67319.20 | 32.283 | 32.276 | 32.376 | 32.346 | 32.332 |
| 681.5181 | 32.351 | 0.879 | 2 | 78253.89 | 61886.29 | 74023.22 | 81999.56 | 71531.17 | 32.351 | 32.340 | 32.443 | 32.406 | 32.387 |
| 571.4479 | 32.216 | 1.079 | 2 | 55120.43 | 42057.73 | 54722.79 | 53133.37 | 51134.51 | 32.216 | 32.212 | 32.309 | 32.286 | 32.278 |
| 549.4341 | 32.216 | 1.146 | 2 | 40206.44 | 36343.37 | 42586.65 | 44967.21 | 44538.88 | 32.216 | 32.212 | 32.309 | 32.286 | 32.278 |
| 703.5339 | 32.418 | 1.146 | 2 | 80279.36 | 60640.08 | 69169.68 | 75640.27 | 67409.19 | 32.418 | 32.404 | 32.511 | 32.467 | 32.441 |
| 527.42 | 32.149 | 0.946 | 2 | 33720.64 | 23439.49 | 27400.31 | 32273.41 | 29468.34 | 32.149 | 32.148 | 32.241 | 32.225 | 32.223 |
| 725.5471 | 32.418 | 0.744 | 2 | 46527.28 | 38802.53 | 43252.91 | 49502.39 | 47137.49 | 32.418 | 32.404 | 32.511 | 32.467 | 32.441 |
| 505.4062 | 32.149 | 0.674 | 2 | 18031.63 | 15215.96 | 17339.12 | 18115.26 | 17887.61 | 32.149 | 32.148 | 32.241 | 32.225 | 32.223 |
| 747.5613 | 32.418 | 0.475 | 2 | 21882.50 | 19172.58 | 22106.01 | 24988.33 | 21334.74 | 32.418 | 32.404 | 32.511 | 32.467 | 32.441 |
| 579.4456 | 29.997 | 0.531 | 2 | 13792.26 | 11058.62 | 12324.22 | 10185.95 | 10885.83 | 29.997 | 30.056 | 30.095 | 30.061 | 30.059 |
| 483.3922 | 32.081 | 0.538 | 2 | 8320.90 | 6827.74 | 7289.89 | 9466.19 | 7959.34 | 32.081 | 32.084 | 32.174 | 32.127 | 32.158 |
| 557.4315 | 29.865 | 0.464 | 2 | 11195.72 | 7505.80 | 10420.83 | 10904.17 | 9422.82 | 29.865 | 29.934 | 29.960 | 29.931 | 29.928 |
| 623.4737 | 30.132 | 0.540 | 2 | 14994.75 | 13420.90 | 12840.42 | 13244.72 | 14297.62 | 30.132 | 30.177 | 30.230 | 30.192 | 30.190 |
| 601.4598 | 30.065 | 0.538 | 2 | 12383.94 | 12635.61 | 14659.69 | 13541.22 | 14250.50 | 30.065 | 30.116 | 30.162 | 30.127 | 30.124 |
| 645.4879 | 30.197 | 0.338 | 2 | 14915.40 | 13813.61 | 16110.67 | 10648.83 | 12787.44 | 30.197 | 30.242 | 30.297 | 30.257 | 30.255 |
| 535.4177 | 29.799 | 0.464 | 2 | 9350.02 | 7021.29 | 7744.54 | 7796.76 | 7653.47 | 29.799 | 29.865 | 29.893 | 29.865 | 29.863 |
| 667.5018 | 30.264 | 0.405 | 2 | 16288.18 | 12110.31 | 13133.84 | 12879.31 | 11899.28 | 30.264 | 30.320 | 30.364 | 30.323 | 30.321 |
| 332.802 | 33.228 | 0.134 | 2 | 6997.25 | 632.70 | 0.00 | 122.29 | 163.77 | 33.228 | 33.233 | 33.314 | 33.298 | 33.260 |
| 325.7939 | 32.960 | 0.134 | 2 | 5999.44 | 267.49 | 138.03 | 129.53 | 0.00 | 32.960 | 32.960 | 33.046 | 33.018 | 32.976 |
| 769.5756 | 32.418 | 0.338 | 2 | 8658.53 | 9534.59 | 9511.62 | 9570.94 | 11479.14 | 32.418 | 32.404 | 32.511 | 32.467 | 32.441 |
| 319.316 | 33.896 | 0.267 | 2 | 773.24 | 1338.31 | 1601.25 | 1616.30 | 1435.13 | 33.896 | 33.912 | 33.986 | 33.958 | 33.926 |
| 689.5156 | 30.333 | 0.340 | 2 | 12898.35 | 9541.09 | 10584.42 | 8554.79 | 9821.96 | 30.333 | 30.404 | 30.432 | 30.388 | 30.386 |
| 513.4035 | 29.734 | 0.397 | 2 | 7962.66 | 5050.83 | 6607.53 | 5451.01 | 4834.30 | 29.734 | 29.793 | 29.827 | 29.800 | 29.798 |
| 333.3321 | 34.299 | 0.336 | 2 | 864.18 | 1084.99 | 1461.25 | 1289.31 | 1354.98 | 34.299 | 34.320 | 34.388 | 34.353 | 34.321 |
| 334.3217 | 33.830 | 0.270 | 2 | 704.70 | 969.44 | 1392.88 | 1056.89 | 1310.62 | 33.830 | 33.844 | 33.920 | 33.866 | 33.871 |
| 546.392 | 24.406 | 0.606 | 2 | 6948.89 | 7368.71 | 5254.05 | 4818.06 | 4666.68 | 24.406 | 24.426 | 24.465 | 24.489 | 24.440 |
| 502.3643 | 24.139 | 0.467 | 2 | 5280.04 | 5566.87 | 5977.22 | 5481.34 | 4659.59 | 24.139 | 24.153 | 24.199 | 24.229 | 24.211 |
| 524.3781 | 24.272 | 0.467 | 2 | 5725.37 | 7694.27 | 6372.62 | 6105.15 | 6500.01 | 24.272 | 24.290 | 24.330 | 24.359 | 24.325 |
| 491.3899 | 29.601 | 0.333 | 2 | 4420.51 | 2395.54 | 2664.66 | 2782.95 | 3149.76 | 29.601 | 29.653 | 29.694 | 29.669 | 29.667 |
| 628.9766 | 32.351 | 0.337 | 2 | 3719.39 | 4906.42 | 3754.67 | 4081.11 | 3632.90 | 32.351 | 32.340 | 32.443 | 32.406 | 32.387 |
| 562.9341 | 32.216 | 0.404 | 2 | 2798.26 | 3315.05 | 2844.20 | 3881.47 | 3141.42 | 32.216 | 32.212 | 32.309 | 32.286 | 32.278 |
| 606.9628 | 32.283 | 0.337 | 2 | 2143.03 | 3898.61 | 3828.99 | 3976.05 | 2586.26 | 32.283 | 32.276 | 32.376 | 32.346 | 32.332 |
| 650.9906 | 32.351 | 0.337 | 2 | 2367.25 | 3805.18 | 3380.55 | 2036.20 | 4303.49 | 32.351 | 32.340 | 32.443 | 32.406 | 32.387 |
| 683.9962 | 32.351 | 0.202 | 2 | 1950.06 | 1992.84 | 2207.70 | 3286.25 | 2451.66 | 32.351 | 32.340 | 32.443 | 32.406 | 32.387 |
| 568.4063 | 24.541 | 0.337 | 2 | 2893.92 | 5412.71 | 4761.11 | 3501.36 | 3923.36 | 24.541 | 24.562 | 24.600 | 24.644 | 24.596 |
| 348.3378 | 34.165 | 0.269 | 2 | 433.72 | 866.71 | 817.72 | 870.57 | 842.59 | 34.165 | 34.183 | 34.255 | 34.233 | 34.189 |
| 584.9484 | 32.283 | 0.337 | 2 | 4595.11 | 3045.70 | 4947.84 | 2785.94 | 3548.75 | 32.283 | 32.276 | 32.376 | 32.346 | 32.332 |
| 802.3012 | 34.030 | 0.403 | 3 | 30840.18 | 14465.07 | 13798.31 | 12499.74 | 13415.32 | 34.030 | 34.049 | 34.121 | 34.114 | 34.057 |
| 461.3787 | 32.081 | 0.336 | 2 | 1867.00 | 1298.47 | 2096.96 | 1808.73 | 2132.38 | 32.081 | 32.084 | 32.174 | 32.127 | 32.158 |
| 617.9538 | 32.283 | 0.270 | 2 | 1941.06 | 1716.23 | 2161.54 | 3683.47 | 2703.12 | 32.283 | 32.276 | 32.376 | 32.346 | 32.332 |
| 595.9394 | 32.283 | 0.202 | 2 | 1396.48 | 2045.06 | 1787.94 | 2349.88 | 1899.37 | 32.283 | 32.276 | 32.376 | 32.346 | 32.332 |
| 791.589 | 32.485 | 0.271 | 2 | 6803.52 | 8065.43 | 6928.79 | 5537.54 | 3565.10 | 32.485 | 32.473 | 32.578 | 32.529 | 32.496 |
| 711.53 | 30.403 | 0.273 | 2 | 5950.06 | 5350.98 | 6091.66 | 6889.48 | 4079.06 | 30.403 | 30.486 | 30.499 | 30.454 | 30.452 |
| 673.0046 | 32.418 | 0.269 | 2 | 3366.25 | 3513.59 | 3584.72 | 5233.62 | 3564.09 | 32.418 | 32.404 | 32.511 | 32.467 | 32.441 |
| 540.9207 | 32.216 | 0.269 | 2 | 1472.00 | 1437.71 | 1878.83 | 2083.41 | 1507.63 | 32.216 | 32.212 | 32.309 | 32.286 | 32.278 |
| 573.9257 | 32.216 | 0.270 | 2 | 1641.89 | 1448.24 | 1082.31 | 1955.68 | 1360.43 | 32.216 | 32.212 | 32.309 | 32.286 | 32.278 |
| 1222.552 | 17.498 | 0.269 | 8 | 0.00 | 43816.56 | 0.00 | 9284.97 | 3283.08 | 17.498 | 17.494 | 17.553 | 17.605 | 17.594 |
| 1245.145 | 25.210 | 0.202 | 2 | 18303.47 | 8658.81 | 5893.86 | 4190.48 | 0.00 | 25.210 | 25.243 | 25.269 | 25.349 | 25.308 |
| 661.4893 | 33.830 | 0.270 | 14 | 1045.59 | 3484.32 | 5481.59 | 3542.37 | 3073.13 | 33.830 | 33.844 | 33.920 | 33.866 | 33.871 |
| 1452.524 | 25.277 | 0.136 | 8 | 3763.52 | 2564.84 | 11041.72 | 0.00 | 5634.12 | 25.277 | 25.310 | 25.337 | 25.414 | 25.376 |
| 401.7857 | 33.497 | 0.333 | 2 | 198.89 | 1595.75 | 1009.97 | 1238.45 | 749.19 | 33.497 | 33.504 | 33.585 | 33.576 | 33.598 |
| 698.582 | 34.030 | 0.269 | 13 | 2330.24 | 1049.39 | 1712.98 | 2067.70 | 1848.79 | 34.030 | 34.049 | 34.121 | 34.114 | 34.057 |
| 1412.815 | 25.210 | 0.133 | 6 | 34473.26 | 16262.18 | 15176.44 | 19860.74 | 28210.71 | 25.210 | 25.243 | 25.269 | 25.349 | 25.308 |
| 802.3015 | 20.518 | 0.269 | 5 | 14511.33 | 6689.27 | 9713.37 | 10324.14 | 9309.27 | 20.518 | 20.575 | 20.573 | 20.610 | 20.636 |
| 469.3444 | 34.367 | 0.270 | 10 | 634.29 | 1889.90 | 2119.48 | 2086.46 | 2517.79 | 34.367 | 34.390 | 34.455 | 34.413 | 34.387 |
| 518.9065 | 32.216 | 0.202 | 2 | 1503.29 | 1863.28 | 438.30 | 571.46 | 1006.21 | 32.216 | 32.212 | 32.309 | 32.286 | 32.278 |
| 480.3503 | 23.872 | 0.332 | 2 | 2381.53 | 3139.38 | 2700.10 | 1982.86 | 2078.93 | 23.872 | 23.885 | 23.928 | 23.968 | 23.969 |
| 586.4662 | 33.562 | 0.266 | 11 | 2970.62 | 2256.68 | 3415.49 | 2855.77 | 2924.99 | 33.562 | 33.571 | 33.650 | 33.636 | 33.653 |
| 639.9685 | 32.351 | 0.270 | 2 | 2287.15 | 2376.46 | 1252.72 | 2886.08 | 2031.58 | 32.351 | 32.340 | 32.443 | 32.406 | 32.387 |
| 590.42 | 24.743 | 0.269 | 2 | 1302.17 | 3498.68 | 1694.33 | 1598.08 | 2515.38 | 24.743 | 24.766 | 24.800 | 24.879 | 24.832 |
| 813.6044 | 32.485 | 0.271 | 2 | 4298.73 | 3545.89 | 3613.33 | 2658.92 | 4071.78 | 32.485 | 32.473 | 32.578 | 32.529 | 32.496 |
| 546.4544 | 32.553 | 0.205 | 18 | 801.63 | 1638.93 | 1649.51 | 656.59 | 359.09 | 32.553 | 32.542 | 32.645 | 32.596 | 32.551 |
| 854.6181 | 34.501 | 0.271 | 14 | 5043.57 | 5264.73 | 1938.40 | 1864.14 | 5467.83 | 34.501 | 34.525 | 34.588 | 34.532 | 34.519 |
| 1161.954 | 25.210 | 0.133 | 4 | 1059.33 | 1717.11 | 4293.10 | 2337.76 | 5447.02 | 25.210 | 25.243 | 25.269 | 25.349 | 25.308 |
| 1064.984 | 16.896 | 0.135 | 5 | 0.00 | 2908.05 | 0.00 | 4528.85 | 1451.67 | 16.896 | 16.852 | 16.949 | 16.997 | 16.975 |
| 706.0097 | 32.351 | 0.135 | 2 | 549.68 | 1354.64 | 1697.71 | 0.00 | 2234.10 | 32.351 | 32.340 | 32.443 | 32.406 | 32.387 |
| 661.9831 | 32.351 | 0.135 | 2 | 3242.00 | 2468.48 | 1578.54 | 2703.89 | 3279.64 | 32.351 | 32.340 | 32.443 | 32.406 | 32.387 |
| 1260.559 | 16.963 | 0.135 | 7 | 0.00 | 9434.96 | 0.00 | 0.00 | 0.00 | 16.963 | 16.925 | 17.017 | 17.064 | 17.044 |
| 552.4133 | 32.216 | 0.202 | 11 | 0.00 | 505.95 | 315.96 | 0.00 | 557.69 | 32.216 | 32.212 | 32.309 | 32.286 | 32.278 |
| 1276.89 | 25.145 | 0.133 | 3 | 1961.86 | 6460.42 | 3490.65 | 11026.89 | 6965.48 | 25.145 | 25.176 | 25.202 | 25.284 | 25.241 |
| 1089.076 | 25.210 | 0.133 | 3 | 38.14 | 5415.59 | 4540.32 | 682.89 | 0.84 | 25.210 | 25.243 | 25.269 | 25.349 | 25.308 |
| 1270.474 | 21.253 | 0.134 | 2 | 0.00 | 1246.55 | 0.00 | 0.00 | 9407.74 | 21.253 | 21.251 | 21.310 | 21.302 | 21.357 |
| 1162.48 | 21.994 | 0.134 | 13 | 0.00 | 0.00 | 0.00 | 0.00 | 5650.66 | 21.994 | 22.032 | 22.048 | 22.046 | 22.139 |
| 1567.426 | 32.216 | 0.135 | 8 | 0.00 | 0.00 | 0.00 | 6610.38 | 0.00 | 32.216 | 32.212 | 32.309 | 32.286 | 32.278 |
| 953.3961 | 20.720 | 0.135 | 7 | 0.00 | 3349.66 | 648.74 | 0.00 | 0.00 | 20.720 | 20.777 | 20.774 | 20.799 | 20.833 |
| 1396 | 21.859 | 0.135 | 2 | 0.00 | 6108.72 | 0.00 | 35.97 | 0.00 | 21.859 | 21.916 | 21.913 | 21.885 | 21.959 |
| 1311.893 | 25.277 | 0.136 | 20 | 0.00 | 2457.36 | 1890.56 | 0.00 | 3237.08 | 25.277 | 25.310 | 25.337 | 25.414 | 25.376 |
| 1420.535 | 19.379 | 0.135 | 6 | 0.00 | 3209.06 | 4022.02 | 0.00 | 2043.86 | 19.379 | 19.406 | 19.434 | 19.495 | 19.512 |
| 1213.392 | 20.787 | 0.135 | 5 | 0.34 | 11219.01 | 1076.95 | 0.00 | 0.00 | 20.787 | 20.835 | 20.841 | 20.862 | 20.899 |
| 469.3761 | 29.534 | 0.135 | 2 | 877.89 | 1046.57 | 602.64 | 751.63 | 701.01 | 29.534 | 29.583 | 29.627 | 29.604 | 29.601 |
| 1093.438 | 32.149 | 0.134 | 4 | 0.00 | 0.00 | 0.00 | 0.00 | 4529.95 | 32.149 | 32.148 | 32.241 | 32.225 | 32.223 |
| 918.6971 | 21.657 | 0.134 | 3 | 0.00 | 108.86 | 0.00 | 2306.43 | 0.00 | 21.657 | 21.707 | 21.711 | 21.679 | 21.690 |
| 1336.212 | 20.787 | 0.135 | 6 | 991.67 | 5476.34 | 0.00 | 0.00 | 0.00 | 20.787 | 20.835 | 20.841 | 20.862 | 20.899 |
| 1015.689 | 21.724 | 0.135 | 9 | 0.00 | 0.00 | 0.00 | 2699.32 | 0.00 | 21.724 | 21.785 | 21.778 | 21.742 | 21.764 |
| 1389.738 | 20.787 | 0.135 | 2 | 0.00 | 4862.09 | 0.00 | 0.00 | 0.00 | 20.787 | 20.835 | 20.841 | 20.862 | 20.899 |
| 1292.439 | 29.865 | 0.131 | 4 | 0.00 | 4707.34 | 0.00 | 0.00 | 0.00 | 29.865 | 29.934 | 29.960 | 29.931 | 29.928 |
| 869.8978 | 21.657 | 0.134 | 3 | 0.00 | 789.43 | 520.53 | 3437.14 | 0.00 | 21.657 | 21.707 | 21.711 | 21.679 | 21.690 |
| 1358.967 | 21.320 | 0.135 | 17 | 0.00 | 143.25 | 0.00 | 0.00 | 3030.74 | 21.320 | 21.324 | 21.377 | 21.364 | 21.409 |
| 1014.563 | 21.657 | 0.134 | 10 | 0.00 | 41.81 | 276.00 | 3038.28 | 0.00 | 21.657 | 21.707 | 21.711 | 21.679 | 21.690 |
| 960.3939 | 21.859 | 0.135 | 8 | 0.00 | 1634.34 | 0.00 | 272.02 | 0.00 | 21.859 | 21.916 | 21.913 | 21.885 | 21.959 |
| 1303.59 | 19.447 | 0.135 | 2 | 0.00 | 3110.91 | 0.00 | 2129.39 | 0.00 | 19.447 | 19.475 | 19.499 | 19.563 | 19.580 |
| 2098.386 | 23.674 | 0.133 | 12 | 0.00 | 0.00 | 0.00 | 6836.82 | 3134.27 | 23.674 | 23.686 | 23.725 | 23.774 | 23.762 |
| 1163.469 | 21.724 | 0.135 | 10 | 0.00 | 0.00 | 0.00 | 4314.84 | 3166.52 | 21.724 | 21.785 | 21.778 | 21.742 | 21.764 |
| 867.2157 | 21.657 | 0.134 | 2 | 0.00 | 0.00 | 0.00 | 2048.30 | 0.00 | 21.657 | 21.707 | 21.711 | 21.679 | 21.690 |
| 962.8974 | 31.141 | 0.133 | 17 | 0.00 | 0.00 | 0.00 | 1520.01 | 0.00 | 31.141 | 31.188 | 31.236 | 31.242 | 31.245 |
| 820.8829 | 21.657 | 0.134 | 3 | 0.00 | 0.00 | 0.00 | 1844.70 | 0.00 | 21.657 | 21.707 | 21.711 | 21.679 | 21.690 |
| 1252.014 | 25.210 | 0.133 | 11 | 0.00 | 0.00 | 2977.73 | 0.00 | 3220.08 | 25.210 | 25.243 | 25.269 | 25.349 | 25.308 |
| 1281.304 | 20.720 | 0.135 | 2 | 0.00 | 4682.11 | 0.00 | 0.00 | 0.00 | 20.720 | 20.777 | 20.774 | 20.799 | 20.833 |
| 1004.426 | 21.724 | 0.135 | 3 | 0.00 | 366.32 | 0.00 | 3795.04 | 0.00 | 21.724 | 21.785 | 21.778 | 21.742 | 21.764 |
| 1537.138 | 28.802 | 0.131 | 11 | 0.00 | 0.00 | 0.00 | 0.00 | 3765.66 | 28.802 | 28.852 | 28.890 | 28.881 | 28.887 |
| 1208.768 | 7.198 | 0.133 | 2 | 0.00 | 5013.25 | 0.00 | 0.00 | 0.00 | 7.198 | 7.069 | 7.243 | 7.267 | 7.270 |
| 1566.589 | 20.720 | 0.135 | 2 | 0.00 | 8497.95 | 0.00 | 0.00 | 0.00 | 20.720 | 20.777 | 20.774 | 20.799 | 20.833 |
| 734.0469 | 30.470 | 0.202 | 2 | 1122.89 | 1352.85 | 858.01 | 0.00 | 0.00 | 30.470 | 30.552 | 30.566 | 30.519 | 30.518 |
| 1313.936 | 21.320 | 0.135 | 20 | 0.00 | 0.00 | 0.00 | 0.00 | 2759.23 | 21.320 | 21.324 | 21.377 | 21.364 | 21.409 |
| 797.6879 | 31.409 | 0.135 | 6 | 0.00 | 1691.69 | 0.00 | 0.00 | 0.00 | 31.409 | 31.444 | 31.504 | 31.484 | 31.505 |
| 795.9906 | 21.724 | 0.135 | 4 | 0.00 | 0.00 | 0.00 | 1473.86 | 0.00 | 21.724 | 21.785 | 21.778 | 21.742 | 21.764 |
| 1468.103 | 31.341 | 0.133 | 2 | 0.00 | 8749.77 | 3230.81 | 0.00 | 0.00 | 31.341 | 31.380 | 31.436 | 31.423 | 31.440 |
| 811.4185 | 22.259 | 0.135 | 5 | 0.00 | 1357.95 | 0.00 | 0.00 | 0.00 | 22.259 | 22.264 | 22.317 | 22.368 | 22.377 |
| 1244.863 | 28.802 | 0.131 | 15 | 0.00 | 0.00 | 0.00 | 626.57 | 4671.98 | 28.802 | 28.852 | 28.890 | 28.881 | 28.887 |
| 1297.119 | 20.720 | 0.135 | 19 | 0.00 | 3172.08 | 0.00 | 0.00 | 0.00 | 20.720 | 20.777 | 20.774 | 20.799 | 20.833 |
| 1404.932 | 29.000 | 0.135 | 12 | 0.00 | 4107.11 | 0.00 | 0.00 | 0.00 | 29.000 | 29.039 | 29.092 | 29.077 | 29.078 |
| 1116.605 | 21.320 | 0.202 | 18 | 0.00 | 1969.83 | 0.00 | 0.00 | 2538.00 | 21.320 | 21.324 | 21.377 | 21.364 | 21.409 |
| 1113.789 | 28.205 | 0.131 | 10 | 0.00 | 3877.65 | 0.00 | 0.00 | 0.00 | 28.205 | 28.297 | 28.281 | 28.292 | 28.338 |
| 1482.733 | 32.081 | 0.134 | 16 | 0.00 | 0.00 | 0.00 | 2444.68 | 3217.17 | 32.081 | 32.084 | 32.174 | 32.127 | 32.158 |
| 1272.878 | 21.859 | 0.135 | 6 | 1780.17 | 3307.26 | 0.00 | 0.00 | 0.00 | 21.859 | 21.916 | 21.913 | 21.885 | 21.959 |
| 1605.481 | 21.994 | 0.134 | 9 | 0.00 | 0.00 | 0.00 | 0.00 | 3736.82 | 21.994 | 22.032 | 22.048 | 22.046 | 22.139 |
| 1398.653 | 31.341 | 0.133 | 11 | 0.00 | 3273.26 | 1462.20 | 0.00 | 0.00 | 31.341 | 31.380 | 31.436 | 31.423 | 31.440 |
| 837.5022 | 21.657 | 0.134 | 2 | 0.00 | 794.41 | 0.00 | 1793.52 | 235.84 | 21.657 | 21.707 | 21.711 | 21.679 | 21.690 |
| 1425.858 | 21.724 | 0.135 | 9 | 0.00 | 0.00 | 2037.33 | 5685.49 | 1345.46 | 21.724 | 21.785 | 21.778 | 21.742 | 21.764 |
| 1416.292 | 20.787 | 0.135 | 15 | 0.00 | 2766.53 | 1756.61 | 0.00 | 0.00 | 20.787 | 20.835 | 20.841 | 20.862 | 20.899 |
| 1439.95 | 22.259 | 0.135 | 18 | 0.00 | 2273.04 | 0.00 | 0.00 | 0.00 | 22.259 | 22.264 | 22.317 | 22.368 | 22.377 |
| 1181.466 | 21.320 | 0.135 | 8 | 0.00 | 1515.54 | 0.00 | 0.00 | 3898.76 | 21.320 | 21.324 | 21.377 | 21.364 | 21.409 |
| 971.3237 | 21.657 | 0.134 | 18 | 0.00 | 429.45 | 0.00 | 2261.31 | 0.00 | 21.657 | 21.707 | 21.711 | 21.679 | 21.690 |
| 1205.143 | 21.253 | 0.134 | 19 | 0.00 | 0.00 | 0.00 | 0.00 | 1736.76 | 21.253 | 21.251 | 21.310 | 21.302 | 21.357 |
| 1216.083 | 28.933 | 0.133 | 7 | 0.00 | 3036.36 | 0.00 | 0.00 | 0.00 | 28.933 | 28.977 | 29.024 | 29.011 | 29.013 |
| 1412.571 | 29.865 | 0.131 | 12 | 0.00 | 3345.95 | 0.00 | 0.00 | 0.00 | 29.865 | 29.934 | 29.960 | 29.931 | 29.928 |
| 843.2562 | 21.657 | 0.134 | 3 | 0.00 | 0.00 | 0.00 | 4047.74 | 795.44 | 21.657 | 21.707 | 21.711 | 21.679 | 21.690 |
| 783.9411 | 21.657 | 0.134 | 9 | 0.00 | 0.00 | 0.00 | 1537.09 | 0.00 | 21.657 | 21.707 | 21.711 | 21.679 | 21.690 |
| 950.2057 | 21.724 | 0.135 | 16 | 0.00 | 0.26 | 0.00 | 3102.46 | 0.00 | 21.724 | 21.785 | 21.778 | 21.742 | 21.764 |
| 1046.669 | 22.259 | 0.135 | 18 | 838.82 | 1664.48 | 0.00 | 1063.27 | 0.00 | 22.259 | 22.264 | 22.317 | 22.368 | 22.377 |
| 1192.757 | 20.787 | 0.135 | 17 | 0.00 | 3207.40 | 0.00 | 0.00 | 1164.10 | 20.787 | 20.835 | 20.841 | 20.862 | 20.899 |
| 1052.436 | 20.787 | 0.135 | 20 | 808.35 | 1964.78 | 0.00 | 0.00 | 0.00 | 20.787 | 20.835 | 20.841 | 20.862 | 20.899 |
| 996.9377 | 28.270 | 0.131 | 3 | 0.00 | 2300.34 | 0.00 | 0.00 | 0.00 | 28.270 | 28.358 | 28.349 | 28.357 | 28.399 |
| 1384.957 | 28.933 | 0.133 | 12 | 0.00 | 4044.39 | 0.00 | 804.70 | 0.00 | 28.933 | 28.977 | 29.024 | 29.011 | 29.013 |
| 1353.284 | 30.470 | 0.135 | 11 | 0.00 | 2891.43 | 0.00 | 0.00 | 0.00 | 30.470 | 30.552 | 30.566 | 30.519 | 30.518 |
| 1245.936 | 21.657 | 0.134 | 14 | 0.00 | 0.00 | 0.00 | 2033.72 | 0.00 | 21.657 | 21.707 | 21.711 | 21.679 | 21.690 |
| 1473.342 | 21.253 | 0.134 | 19 | 0.00 | 0.00 | 0.00 | 0.00 | 3030.04 | 21.253 | 21.251 | 21.310 | 21.302 | 21.357 |
| 1221.121 | 20.720 | 0.135 | 19 | 0.00 | 919.51 | 0.00 | 0.00 | 0.00 | 20.720 | 20.777 | 20.774 | 20.799 | 20.833 |
| 800.8882 | 20.787 | 0.135 | 12 | 0.00 | 1085.61 | 0.00 | 0.00 | 0.00 | 20.787 | 20.835 | 20.841 | 20.862 | 20.899 |
| 2005.284 | 20.720 | 0.135 | 9 | 0.00 | 4706.10 | 0.00 | 0.00 | 0.00 | 20.720 | 20.777 | 20.774 | 20.799 | 20.833 |
| 1714.458 | 20.720 | 0.135 | 10 | 0.00 | 3603.94 | 0.00 | 0.00 | 0.00 | 20.720 | 20.777 | 20.774 | 20.799 | 20.833 |
| 1236.973 | 32.283 | 0.135 | 3 | 0.00 | 3724.24 | 0.00 | 0.00 | 0.00 | 32.283 | 32.276 | 32.376 | 32.346 | 32.332 |
| 1513.448 | 28.270 | 0.131 | 10 | 0.00 | 3093.99 | 0.00 | 0.00 | 0.00 | 28.270 | 28.358 | 28.349 | 28.357 | 28.399 |
| 1304.315 | 31.341 | 0.133 | 14 | 2065.73 | 1771.28 | 0.00 | 0.00 | 0.00 | 31.341 | 31.380 | 31.436 | 31.423 | 31.440 |
| 971.2511 | 29.865 | 0.131 | 7 | 0.00 | 1121.13 | 0.00 | 0.00 | 0.00 | 29.865 | 29.934 | 29.960 | 29.931 | 29.928 |
| 997.322 | 22.259 | 0.135 | 3 | 0.00 | 1788.54 | 0.00 | 0.00 | 0.00 | 22.259 | 22.264 | 22.317 | 22.368 | 22.377 |
| 956.3617 | 20.787 | 0.135 | 5 | 0.00 | 2684.78 | 0.00 | 0.00 | 0.00 | 20.787 | 20.835 | 20.841 | 20.862 | 20.899 |
| 1316.245 | 21.724 | 0.135 | 11 | 0.00 | 0.00 | 0.00 | 3012.66 | 0.00 | 21.724 | 21.785 | 21.778 | 21.742 | 21.764 |
| 1375.842 | 20.720 | 0.135 | 17 | 0.00 | 2178.76 | 0.00 | 0.00 | 0.00 | 20.720 | 20.777 | 20.774 | 20.799 | 20.833 |
| 1779.183 | 31.409 | 0.135 | 9 | 0.00 | 2896.74 | 0.00 | 0.00 | 0.00 | 31.409 | 31.444 | 31.504 | 31.484 | 31.505 |
| 1438.395 | 20.720 | 0.135 | 9 | 0.00 | 3798.72 | 0.00 | 0.00 | 0.00 | 20.720 | 20.777 | 20.774 | 20.799 | 20.833 |
| 1220.232 | 20.787 | 0.135 | 4 | 18.85 | 2913.12 | 0.00 | 0.00 | 0.00 | 20.787 | 20.835 | 20.841 | 20.862 | 20.899 |
| 1305.044 | 32.216 | 0.135 | 13 | 0.00 | 2956.20 | 0.00 | 0.00 | 0.00 | 32.216 | 32.212 | 32.309 | 32.286 | 32.278 |
| 1552.844 | 31.341 | 0.133 | 11 | 0.00 | 3823.35 | 0.00 | 3083.03 | 0.00 | 31.341 | 31.380 | 31.436 | 31.423 | 31.440 |
| 982.1032 | 22.259 | 0.135 | 3 | 0.00 | 4052.23 | 683.53 | 0.00 | 443.16 | 22.259 | 22.264 | 22.317 | 22.368 | 22.377 |
| 1169.702 | 20.720 | 0.135 | 15 | 0.00 | 2865.93 | 566.25 | 0.00 | 0.00 | 20.720 | 20.777 | 20.774 | 20.799 | 20.833 |
| 1094.67 | 22.191 | 0.133 | 2 | 2011.54 | 2356.81 | 24.74 | 0.00 | 0.00 | 22.191 | 22.205 | 22.250 | 22.287 | 22.317 |
| 1813.41 | 30.470 | 0.135 | 11 | 0.00 | 3783.79 | 0.00 | 0.00 | 0.00 | 30.470 | 30.552 | 30.566 | 30.519 | 30.518 |
| 1288.052 | 20.787 | 0.135 | 11 | 0.00 | 3111.42 | 0.00 | 0.00 | 0.00 | 20.787 | 20.835 | 20.841 | 20.862 | 20.899 |
| 1969.687 | 31.341 | 0.133 | 17 | 0.00 | 3916.57 | 0.00 | 0.00 | 0.00 | 31.341 | 31.380 | 31.436 | 31.423 | 31.440 |
| 1241.583 | 32.216 | 0.135 | 18 | 0.00 | 2331.13 | 0.00 | 0.00 | 0.00 | 32.216 | 32.212 | 32.309 | 32.286 | 32.278 |
| 1039.69 | 22.191 | 0.133 | 4 | 375.78 | 1488.05 | 0.00 | 0.00 | 0.00 | 22.191 | 22.205 | 22.250 | 22.287 | 22.317 |
| 1154.465 | 28.205 | 0.131 | 15 | 0.00 | 1747.61 | 0.00 | 0.00 | 0.00 | 28.205 | 28.297 | 28.281 | 28.292 | 28.338 |
| 1408.574 | 32.216 | 0.135 | 12 | 2126.45 | 2655.85 | 0.00 | 0.00 | 0.00 | 32.216 | 32.212 | 32.309 | 32.286 | 32.278 |
| 866.069 | 21.657 | 0.134 | 2 | 0.00 | 91.36 | 0.00 | 2506.26 | 0.00 | 21.657 | 21.707 | 21.711 | 21.679 | 21.690 |
| 1715.638 | 32.216 | 0.135 | 13 | 0.00 | 3901.60 | 0.00 | 0.00 | 0.00 | 32.216 | 32.212 | 32.309 | 32.286 | 32.278 |
| 1215.061 | 22.191 | 0.133 | 11 | 0.00 | 2344.37 | 0.00 | 0.00 | 0.00 | 22.191 | 22.205 | 22.250 | 22.287 | 22.317 |
| 1244.685 | 32.216 | 0.135 | 2 | 0.00 | 1945.92 | 0.00 | 0.00 | 0.00 | 32.216 | 32.212 | 32.309 | 32.286 | 32.278 |
| 1137.733 | 22.259 | 0.135 | 11 | 0.00 | 2441.72 | 0.00 | 0.00 | 0.00 | 22.259 | 22.264 | 22.317 | 22.368 | 22.377 |
| 1299.908 | 21.320 | 0.135 | 12 | 0.00 | 0.00 | 0.00 | 1925.55 | 4155.67 | 21.320 | 21.324 | 21.377 | 21.364 | 21.409 |
| 951.8954 | 21.657 | 0.134 | 20 | 0.00 | 0.00 | 0.00 | 1633.00 | 0.00 | 21.657 | 21.707 | 21.711 | 21.679 | 21.690 |
| 1463.775 | 32.216 | 0.135 | 12 | 0.00 | 3035.86 | 0.00 | 0.00 | 0.00 | 32.216 | 32.212 | 32.309 | 32.286 | 32.278 |
| 1189.711 | 32.283 | 0.135 | 9 | 0.00 | 3088.48 | 620.97 | 0.00 | 159.05 | 32.283 | 32.276 | 32.376 | 32.346 | 32.332 |
| 1302.26 | 29.865 | 0.131 | 17 | 0.00 | 1680.23 | 0.00 | 0.00 | 0.00 | 29.865 | 29.934 | 29.960 | 29.931 | 29.928 |
| 1505.809 | 20.720 | 0.135 | 15 | 2445.37 | 4449.98 | 6692.08 | 0.00 | 0.00 | 20.720 | 20.777 | 20.774 | 20.799 | 20.833 |
| 1212.033 | 28.205 | 0.131 | 20 | 0.00 | 1795.79 | 0.00 | 0.00 | 0.00 | 28.205 | 28.297 | 28.281 | 28.292 | 28.338 |
| 1557.637 | 28.205 | 0.131 | 13 | 0.00 | 2809.00 | 0.00 | 0.00 | 0.00 | 28.205 | 28.297 | 28.281 | 28.292 | 28.338 |
| 1206.437 | 20.720 | 0.135 | 15 | 0.00 | 1825.34 | 0.00 | 0.00 | 0.00 | 20.720 | 20.777 | 20.774 | 20.799 | 20.833 |
| 1123.427 | 22.259 | 0.135 | 3 | 1337.64 | 2219.22 | 0.00 | 0.00 | 1127.07 | 22.259 | 22.264 | 22.317 | 22.368 | 22.377 |
| 1379.446 | 22.663 | 0.135 | 14 | 0.00 | 0.00 | 0.00 | 0.00 | 2077.69 | 22.663 | 22.671 | 22.722 | 22.739 | 22.734 |
| 579.4346 | 32.216 | 0.135 | 7 | 0.00 | 366.89 | 0.00 | 0.00 | 0.00 | 32.216 | 32.212 | 32.309 | 32.286 | 32.278 |
| 1030.393 | 29.930 | 0.133 | 12 | 0.00 | 1355.15 | 0.00 | 1123.36 | 0.00 | 29.930 | 29.995 | 30.028 | 29.996 | 29.994 |
| 1432.695 | 32.216 | 0.135 | 14 | 0.00 | 1542.38 | 0.00 | 0.00 | 0.00 | 32.216 | 32.212 | 32.309 | 32.286 | 32.278 |
| 1378.455 | 28.270 | 0.131 | 18 | 0.00 | 981.97 | 0.00 | 0.00 | 0.00 | 28.270 | 28.358 | 28.349 | 28.357 | 28.399 |
